# Geographical variation in lung function: Results from the multicentric cross-sectional BOLD study

**DOI:** 10.1080/25310429.2024.2430491

**Published:** 2024-12-06

**Authors:** Peter G.J. Burney, James Potts, Ben Knox-Brown, Gregory Erhabor, Hamid Hacene Cherkaski, Kevin Mortimer, Mahesh Padukudru Anand, David M Mannino, Joao Cardoso, Rana Ahmed, Asma Elsony, Cristina Barbara, Rune Nielsen, Eric Bateman, Stefanni Nonna M Paraguas, Li Cher Loh, Abdul Rashid, Emiel FM Wouters, Frits ME Franssen, Hermínia Brites Dias, Thorarinn Gislason, Mohammed Al Ghobain, Mohammed El Biaze, Dhiraj Agarwal, Sanjay Juvekar, Fatima Rodrigues, Daniel O Obaseki, Parvaiz A. Koul, Imed Harrabi, Asaad A Nafees, Terence Seemungal, Christer Janson, William M Vollmer, Andre FS Amaral, A Sonia Buist

**Affiliations:** a National Heart and Lung Institute, Imperial College London, London, UK; b Department of Medicine, Obafemi Awolowo University/Obafemi Awolowo University Teaching Hospitals Complex, Osun, Nigeria; c Department of Pulmonology, Faculty of Medicine, University Badji Mokhtar, Annaba, Algeria; d Department of Medicine, University of Cambridge, Cambridge, UK; e Department of Respiratory Medicine, Liverpool University Hospitals NHS Foundation Trust, Liverpool, UK; f Department of Respiratory Medicine, JSS Medical College, JSSAHER, Mysuru, India; g Division of Pulmonary and Critical Care Medicine, University of Kentucky, Lexington, KY, USA; h COPD Foundation, Miami, FL, USA; i Pulmonology Department, Centro Hospitalar Universitário de Lisboa Central, Lisboa, Portugal; j NOVA Medical School, Nova University Lisbon, Lisboa, Portugal; k The Epidemiological Laboratory (Epi-Lab), Khartoum, Sudan; l Instituto de Saúde Ambiental, Faculdade de Medicina, Universidade de Lisboa, Lisbon, Portugal; m Serviço de Pneumologia, Centro Hospitalar Universitário Lisboa Norte, Lisbon, Portugal; n Department of Clinical Science, University of Bergen, Bergen, Norway; o Department of Thoracic Medicine, Haukeland University Hospital, Bergen, Norway; p Department of Medicine, University of Cape Town and UCT Lung Institute, Cape Town, South Africa; q Philippine College of Chest Physicians, Manila, Philippines; r Philippine Heart Centre, Manila, Philippines; s Department of Public Health, Royal College of Surgeons in Ireland and University College Dublin Malaysia Campus, Penang, Malaysia; t Faculty of Medicine, Sigmund Freud University, Vienna, Austria; u Department of Respiratory Medicine, Maastricht University Medical Centre, Maastricht, the Netherlands; v Lisbon School of Health Technology, Polytechnic of Lisbon, Lisbon, Portugal; w Faculty of Medicine, University of Iceland, Reykjavík, Iceland; x Department of Sleep, Landspitali - The National University Hospital of Iceland, Reykjavik, Iceland; y Department of Medicine, King Saud bin Abdulaziz University for Health Sciences, Riyadh, Saudi Arabia; z King Abdullah International Medical Research Centre, Riyadh, Saudi Arabia; aa Department of Respiratory Medicine, Faculty of Medicine, Mohammed Ben Abdellah University, Fes, Morocco; bb Vadu Rural Health Program, KEM Hospital Research Centre, Pune, India; cc Institute of Environmental Health, Associate Laboratory TERRA, Lisbon Medical School, Lisbon University, Lisbon, Portugal; dd Faculty of Medicine, University of British Columbia, Vancouver, BC, Canada; ee Department of Pulmonary Medicine, Sheri Kashmir Institute of Medical Sciences, Srinagar, India; ff Ibn El Jazzar Faculty of Medicine of Sousse, University of Sousse, Sousse, Tunisia; gg Department of Community Health Sciences, The Aga Khan University, Karachi, Pakistan; hh Department of Clinical Medical Sciences, The University of The West Indies, St Augustine, Trinidad and Tobago; ii Department of Medical Sciences, Respiratory Allergy and Sleep Research, Uppsala University, Uppsala, Sweden; jj Center for Health Research, Kaiser Permanente Center for Health Research, Portland, OR, USA; kk NIHR Imperial Biomedical Research Centre, London, UK; ll Division of Pulmonary and Critical Care Medicine, Oregon Health and Science University, Portland, OR, USA

**Keywords:** Cross-sectional studies, global health, forced expiratory volume, forced vital capacity, airflow obstruction

## Abstract

Spirometry is used to determine what is “unusual” lung function compared with what is “usual” for healthy non-smokers. This study aimed to investigate regional variation in the forced vital capacity (FVC) and in the forced expiratory volume in one second to FVC ratio (FEV_1_/FVC) using cross-sectional data from all 41 sites of the multinational Burden of Obstructive Lung Disease study. Participants (5,368 men; 9,649 women), aged ≥40 years, had performed spirometry, had never smoked and reported no respiratory symptoms or diagnoses. To identify regions with similar FVC, we conducted a principal component analysis (PCA) on FVC with age, age^2^ and height^2^, separately for men and women. We regressed FVC against age, age^2^ and height^2^, and FEV_1_/FVC against age and height^2^, for each sex and site, stratified by region. Mean age was 54 years (both sexes), and mean height was 1.69 m (men) and 1.61 m (women). The PCA suggested four regions: 1) Europe and richer countries; 2) the Near East; 3) Africa; and 4) the Far East. For the FVC, there was little variation in the coefficients for age, or age^2^, but considerable variation in the constant (men: 2.97 L in the Far East to 4.08 L in Europe; women: 2.44 L in the Far East to 3.24 L in Europe) and the coefficient for height^2^. Regional differences in the constant and coefficients for FEV_1_/FVC were minimal (<1%). The relation of FVC with age, sex and height varies across and within regions. The same is not true for the FEV_1_/FVC ratio.

## SUMMARY AT A GLANCE

The relation of FVC to age and height varies geographically, but there is no geographical variation in the FEV_1_/FVC ratio. These findings may be useful for identifying specific restrictive lung disease, and they do not alter the current advice to use a single global standard when assessing severity of disease.

## Introduction

Until recently, it has been a standard practice to provide separate algorithms to identify normal lung function and specifically spirometry for different ethnic groups,^1,2^ or to provide adjustments.^3^ More recently, this practice has been criticised on three grounds.^4^ First, because ethnic differences are widely misinterpreted as fixed biological properties rather than as the result of more complex origins, including social disadvantage.^5^ Second, because in many places, population mixing makes ethnic affiliations hard to define. And finally, it has been shown that, at least in the USA, the same measure of vital capacity, adjusted for age, sex and height, gives the same prediction of outcome for both European Americans and African Americans.^6–9^ Using separate standards disguises true disadvantages for ethnic groups with lower lung function. More recently, Bowerman et al. have provided a single global algorithm for estimating predicted lung function using the Global Lung Initiative (GLI) database.^10^

It remains the case that lung function varies widely across different countries and even within regions.^11^ This creates problems when distinguishing specific lung pathology from the general disadvantage suffered by a local population. In this case, it is still necessary to take into account the lower (or higher) lung function seen in the local population. The objective of this analysis was to estimate how far spirometric indices, specifically the forced vital capacity (FVC) and ratio of forced expiratory volume in one second (FEV_1_) to FVC (FEV_1_/FVC), vary between and within broad regions and to identify how much overlap there was between these broad regions. We have used the multinational Burden of Obstructive Lung Disease (BOLD) study to identify global patterns of FVC. We have further used regression to assess variation in the coefficients predicting FVC and variation in the coefficients predicting the FEV_1_/FVC ratio, and to estimate the variation both across and within regions. The main point of the analysis is to help clinicians who want to know whether a patient’s lung function is similar to that of someone from the same population who does not smoke, does not have any symptoms and does not have a respiratory diagnosis.

## Methods

The methods of the BOLD study have been published.^12^ The current analysis is of a clustered cross-sectional survey of representative samples drawn from selected populations. Briefly, 41 sites were selected to represent all regions of the world, except Latin America, which had its own study,^13^ Oceania and the richer countries of the Pacific region. One site was excluded because their lung function data were judged unreliable. Within each site, a representative sample of the non-institutionalised population aged 40 years or over was identified. The centres were asked to include a minimum of 600 individuals each to provide an acceptable precision to estimates of prevalence.^12^ In addition to standardised questionnaires, height was measured, and lung function was assessed using the ndd EasyOne spirometer (ndd Medizintechnik AG, Zurich, Switzerland) before and after administration of 200 mcg salbutamol (albuterol) inhaled through a spacer. All recordings from the spirometers were downloaded and assessed for compliance with ATS/ERS standards current at the time. Feedback was sent to all technicians and those who failed to maintain good standards were suspended and retrained.^14^ All participants gave informed consent before taking part, and the protocol was approved by each local ethics committee as well as by the Charing Cross Research Ethics Committee (06/Q0411/97) in London, UK.

Within each site, we selected all healthy, non-smoking participants, defined as having no history of smoking, no respiratory diagnosis (including asthma, emphysema, chronic bronchitis or tuberculosis) and no reported respiratory symptoms (including wheeze, cough, phlegm or breathlessness) with a quality-approved spirometry.

We conducted a principal component analysis (PCA) using data for age, age^2^, height^2^ and FVC, for men and women separately. This reduces the correlations between the variables to a minimum number of uncorrelated values that are themselves without dimension. We estimated the mean values of the first four components for each study site and plotted these.

We regressed the FVC for each region against the same independent variables, age, age^2^ and height^2^, as suggested by Hankinson et al.^1^ using a multi-level model to account for variation across sites. We regressed the FEV_1_/FVC ratio against age and height^2^, as suggested by Kiefer et al.^15^ using a similar multi-level model to account for variation across sites.

We centred the analyses to age 40, the youngest age included in the sample, and height to 1.40 m, to ensure that we included all possible individuals but avoided negative values of height when estimating height^2^.

All analyses were undertaken using Stata 17.0 (StataCorp LP, College Station, TX, USA).

## Results

Table 1 presents the numbers of men and women who had good-quality spirometry and a valid age and height. It also presents the number of healthy, non-smoking men and women seen in each study site. Only a third of the men (5,368/16,104) and just over a half of the women (9,649/18,014) met the definition of “normal”. Table 2 presents the characteristics of the “normal” men and women including the mean and standard deviation for age, height and FVC. Among these “normal” individuals, the mean age ranged from 46 years (Mysore, India) to 65 years (Lisbon, Portugal) in men and from 45 years (Mysore, India) to 65 years (Lisbon, Portugal) in women. Mean height ranged from 1.63 m (Sri Lanka) to 1.80 m (Reykjavik, Iceland) in men and from 1.51 m (Sri Lanka) to 1.65 m (Reykjavik, Iceland) in women. Mean FVC ranged from 2.85 L (Sri Lanka) to 4.92 L (Vancouver, Canada) in men and from 2.05 L (Sri Lanka) to 3.30 L (Salzburg, Austria and Maastricht, Netherlands) in women.

**Table 1.** Numbers in analyses.

	Men			Women		
Site	Valid*	Normal**	(% normal)	Valid*	Normal**	(% normal)
Albania (Tirana)	529	205	38.8	528	435	82.4
Algeria (Annaba)	458	96	21.0	458	350	76.4
Australia (Sydney)	291	65	22.3	294	69	23.5
Austria (Salzburg)	736	152	20.7	613	181	29.5
Benin (Sèmè-Kpodji)	375	323	86.1	491	432	88.0
Cameroon (Limbe)	340	193	56.8	231	175	75.8
Canada (Vancouver)	363	83	22.9	493	114	23.1
China (Guangzhou)	291	53	18.2	309	233	75.4
England (London)	338	45	13.3	359	73	20.3
Estonia (Tartu)	327	70	21.4	319	126	39.5
Germany Hannover)	366	60	16.4	346	76	22.0
Iceland (Reykjavik)	404	74	18.3	356	57	16.0
India (Kashmir)	536	145	27.1	481	328	68.2
India (Mumbai)	315	244	77.5	200	162	81.0
India (Mysore)	395	283	71.6	471	443	94.1
India (Pune)	631	450	71.3	532	488	91.7
Jamaica	312	99	31.7	453	213	47.0
Kyrgyzstan (Chui)	343	59	17.2	681	420	61.7
Kyrgyzstan (Naryn)	425	128	30.1	632	428	67.7
Malawi (Blantyre)	211	121	57.3	313	239	76.4
Malawi (Chikwawa)	324	147	45.4	385	285	74.0
Malaysia (Penang)	374	160	42.8	350	284	81.1
Morocco (Fes)	407	123	30.2	526	338	64.3
Netherlands (Maastricht)	324	54	16.7	305	75	24.6
Nigeria (Ife)	427	293	68.6	708	600	84.7
Norway (Bergen)	348	72	20.7	359	89	24.8
Pakistan (Karachi)	389	170	43.7	547	377	68.9
Philippines (Manila)	385	26	6.8	523	111	21.2
Philippines (Nampicuan/Talugtug)	383	50	13.1	389	184	47.3
Poland (Krakow)	302	35	11.6	301	66	21.9
Portugal (Lisbon)	396	108	27.3	495	227	45.9
Saudi Arabia (Riyadh)	486	193	39.7	379	146	38.5
South Africa (Ravensmead/Uitsig)	335	42	12.5	558	118	21.1
Sri Lanka	548	174	31.8	721	362	50.2
Sudan (Gezeira)	418	177	42.3	387	245	63.3
Sudan (Khartoum)	399	215	53.9	264	198	75.0
Sweden (Uppsala)	306	61	19.9	282	66	23.4
Trinidad and Tobago	576	213	37.0	815	501	61.5
Tunisia (Sousse)	330	42	12.7	384	186	48.4
Turkey (Adana)	425	43	10.1	449	94	20.9
USA (Lexington, KY)	236	22	9.3	327	55	16.8
**Total**	**16,104**	**5,368**	**33.3**	**18,014**	**9,649**	**53.6**

*With good-quality spirometry and valid age and height.

**Valid and never smoked, with no respiratory symptom and with no respiratory diagnosis.

**Table 2.** “Normal” participants, their mean age, mean height and mean FVC by BOLD site.

	Men			Women		
	Age (years)	Height (cm)	FVC* (L)	Age (years)	Height (cm)	FVC* (L)
Site	Mean (SD)	Mean (SD)	Mean (SD)	Mean (SD)	Mean (SD)	Mean (SD)
“Europe”: Mostly high-income countries with predominantly European languages						
Norway (Bergen)	57 (12)	180 (7)	4.76 (0.85)	59 (13)	171 (10)	3.41 (0.64)
Germany (Hannover)	57 (11)	177 (9)	4.75 (0.81)	58 (12)	169 (11)	3.23 (0.73)
Poland (Krakow)	50 (11)	174 (6)	4.81 (0.79)	56 (13)	165 (9)	3.15 (0.61)
USA (Lexington, KY)	56 (11)	178 (6)	4.75 (0.97)	56 (10)	167 (9)	3.13 (0.47)
Portugal (Lisbon)	65 (11)	167 (7)	3.85 (0.87)	64 (12)	159 (9)	2.82 (0.63)
England (London)	56 (12)	175 (7)	4.37 (1.03)	59 (12)	167 (9)	3.02 (0.59)
Netherlands (Maastricht)	53 (11)	178 (7)	4.96 (0.83)	56 (12)	169 (10)	3.28 (0.73)
Iceland (Reykyavik)	53 (12)	180 (7)	5.01 (0.82)	54 (12)	174 (10)	3.36 (0.64)
Austria (Salzburg)	57 (11)	176 (7)	4.68 (0.79)	57 (11)	169 (9)	3.48 (0.65)
Estonia (Tartu)	64 (12)	175 (7)	4.48 (0.80)	63 (12)	167 (9)	3.22 (0.76)
Albania (Tirana)	57 (11)	168 (8)	4.01 (0.82)	54 (10)	162 (8)	3.05 (0.59)
Sweden (Uppsala)	57 (13)	179 (7)	4.75 (0.92)	56 (12)	171 (10)	3.39 (0.64)
Canada (Vancouver)	52 (9)	176 (8)	5.00 (0.96)	53 (10)	167 (11)	3.40 (0.69)
Australia (Sydney)	58 (12)	172 (8)	4.34 (0.91)	59 (13)	165 (10)	3.03 (0.70)
“Near East”: North Africa and Western and Central Asia						
Turkey (Adana)	56 (11)	167 (7)	3.94 (0.80)	56 (12)	157 (9)	2.83 (0.48)
Algeria (Annaba)	53 (11)	171 (8)	4.08 (0.74)	52 (10)	160 (9)	2.87 (0.53)
Morocco (Fes)	57 (11)	168 (7)	3.92 (0.82)	55 (11)	159 (9)	2.91 (0.64)
Kyrgyzstan (Chui)	54 (11)	168 (7)	4.38 (0.74)	52 (9)	158 (8)	2.99 (0.52)
Kyrgyzstan (Naryn)	55 (11)	168 (6)	4.15 (0.77)	53 (10)	158 (8)	3.03 (0.50)
Saudi Arabia (Riyadh)	51 (9)	168 (8)	3.45 (0.79)	50 (8)	163 (9)	2.60 (0.44)
Tunisia (Sousse)	55 (9)	170 (7)	4.06 (0.81)	53 (9)	149 (9)	2.90 (0.64)
“Africa”: Sub-Saharan Africa and West Indies						
Benin (Sèmè-Kpodji)	52 (10)	171 (7)	3.30 (0.64)	51 (10)	165 (8)	2.43 (0.45)
Malawi (Blantyre)	54 (10)	168 (7)	3.50 (0.69)	52 (10)	161 (8)	2.64 (0.50)
South Africa (Ravensmead/Uitsig)	54 (11)	171 (8)	3.79 (0.77)	55 (12)	161 (9)	2.69 (0.64)
Malawi (Chikwawa)	56 (12)	166 (8)	3.65 (0.62)	55 (12)	159 (9)	2.69 (0.46)
Nigeria (Ife)	57 (12)	168 (7)	3.18 (0.67)	56 (12)	162 (8)	2.37 (0.46)
Jamaica	56 (13)	172 (7)	3.58 (0.73)	57 (13)	165 (9)	2.60 (0.58)
Cameroon (Limbe)	51 (11)	168 (8)	3.12 (0.84)	51 (10)	165 (8)	2.54 (0.49)
Trinidad and Tobago	55 (11)	173 (8)	3.38 (0.72)	54 (11)	164 (9)	2.32 (0.54)
Sudan (Gezeira)	55 (11)	170 (8)	3.31 (0.64)	53 (10)	163 (9)	2.41 (0.49)
Sudan (Khartoum)	55 (10)	169 (8)	3.31 (0.74)	53 (10)	165 (9)	2.47 (0.56)
“Far East”: South, South-East and East Asia						
China (Guangzhou)	56 (13)	167 (6)	3.57 (0.82)	53 (11)	157 (8)	2.67 (0.55)
Pakistan (Karachi)	52 (11)	167 (8)	3.17 (0.72)	51 (10)	159 (9)	2.20 (0.57)
Philippines (Manila)	53 (12)	163 (6)	3.11 (0.63)	53 (11)	154 (7)	2.29 (0.44)
India (Mumbai)	51 (9)	166 (6)	3.31 (0.59)	50 (9)	161 (9)	2.38 (0.48)
India (Mysore)	47 (7)	163 (6)	3.11 (0.75)	46 (7)	158 (6)	2.23 (0.46)
Philippines (Nampicuan/Talugtug)	50 (9)	166 (6)	3.48 (0.62)	52 (9)	157 (8)	2.33 (0.51)
Malaysia (Penang)	57 (10)	164 (6)	3.01 (0.55)	55 (10)	157 (8)	2.37 (0.52)
India (Pune)	52 (10)	164 (7)	3.20 (0.55)	52 (10)	158 (9)	2.24 (0.38)
Sri Lanka	49 (11)	166 (7)	3.84 (0.81)	54 (10)	155 (8)	2.06 (0.45)
India (Kashmir)	49 (9)	166 (8)	3.84 (0.72)	50 (9)	158 (9)	2.72 (0.56)
Total	54 (11)	169 (8)	3.64 (0.93)	54 (11)	161 (9)	2.66 (0.65)

”Normal” participants have valid age and height, have never smoked and report no respiratory symptoms or diagnoses. FVC, Forced vital capacity. SD, Standard deviation.

Fieldwork was conducted between 2003 and 2016. The dates of the fieldwork in each study site have been published earlier.^16^

The PCA identified four groups of sites. There was no uncontentious way of labelling these succinctly, but we have labelled as “Europe” the group of countries in Europe and those that appeared in the same group in the PCA, including the USA, Canada and Australia. We labelled as “Near East” those countries in North Africa, Western and Central Asia, as “Africa” countries of sub-Saharan Africa and the two Caribbean sites which appeared in the same group, and as “Far East” countries in South, South-East and Eastern Asia.

**Figure 1.**
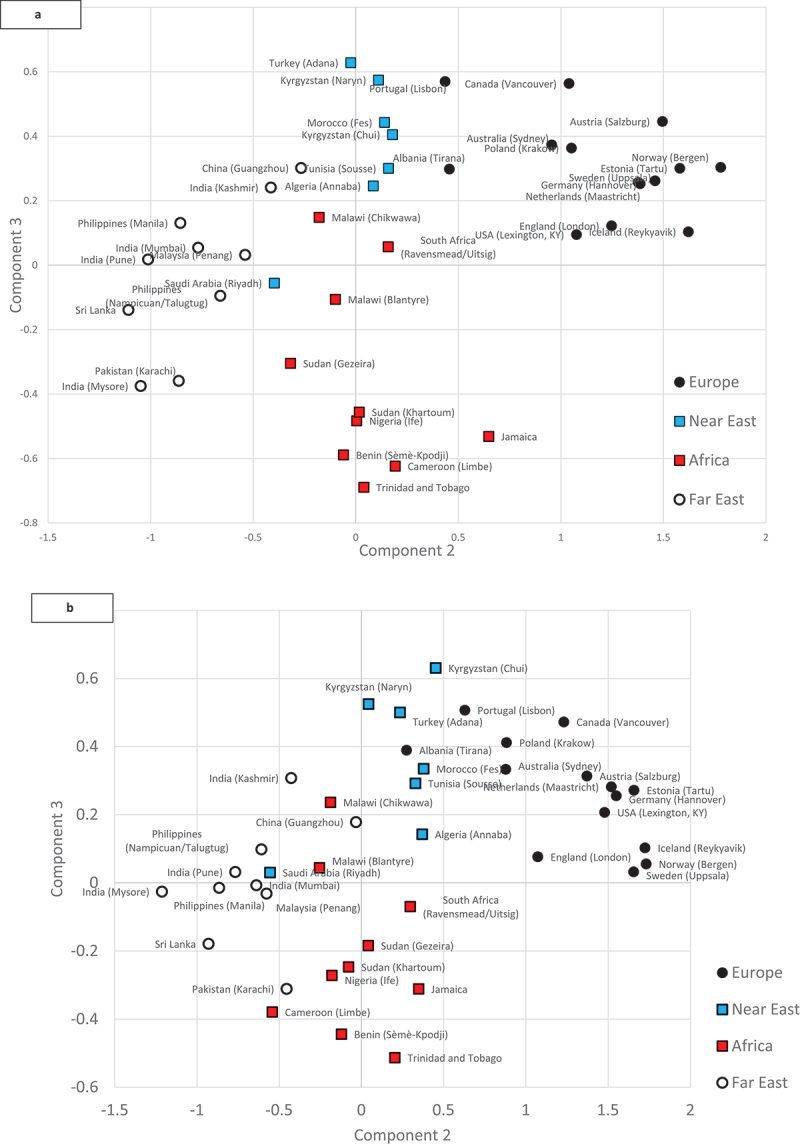
Principal component analysis of FVC, age, age^2^ and height^2^ for women (a) and men (b).

The plot of the third principal component against the second principal component from the PCA for women is shown in Figure 1a. The second principal component clearly separates the “European” and “Far Eastern” sites, with the “Near Eastern” and “African” sites being intermediate between the other two. The third principal component separates the “African and “Near Eastern” sites. There is little overlap between the regions, and the only real exception is Riyadh (Saudi Arabia), which is placed in the Far Eastern group, some way distant from the other “Near Eastern” sites. The plot for men is similar (Figure 1b), with Riyadh (Saudi Arabia) again clearly out of place, as with women. In addition, Tirana (Albania) appears in the “Near Eastern” group, and Guangzhou (China) also seems slightly displaced.

The regression analyses for FVC against age, age^2^ and height^2^ showed relatively little variation across sites in the effect of age or age^2^ on the FVC (Table 3), though the cross-sectional decline with age appears steeper in those in the “European” sites. The effect of height on FVC is less strong in the African Region, with FVC rising less for each extra unit of height than in other regions. Variation in the constant (equivalent to the estimated FVC in a 40-year-old with a height of 1.40 m) is also high. In both men and women, this constant was highest among people in the European region, next highest in the Near East, next in Africa and lowest in the Far East. The “global” values are the values obtained for the dataset as a whole without specifying region.

**Table 3.** Regional variation in forced vital capacity (L), showing regression coefficients from multi-level models with the root mean square error for the estimated value (50th centile) and lower limit of normal (LLN) (5th centile).

Men					
Region	Constant	Constant* (LLN**)	Age	Age^2^	Height^2^
Europe	4.08	3.04	-0.038	0.000132	0.000798
Near East	3.57	2.48	-0.025	0.000018	0.000881
Africa	3.17	2.17	-0.026	0.000131	0.000642
Far East	2.97	2.05	-0.023	0.000063	0.000786
Global	3.48	2.22	-0.025	0.000014	0.000749
Women					
Region	Constant	Constant* (LLN**)	Age	Age^2^	Height^2^
Europe	3.24	2.47	-0.032	0.000061	0.000920
Near East	2.87	2.14	-0.026	0.000088	0.001026
Africa	2.44	1.69	-0.018	0.000032	0.000658
Far East	2.36	1.62	-0.025	0.000215	0.000906
Global	2.74	1.80	-0.021	-0.000033	0.000849

*Constant for person of 1.40 m and 40 years old; **lower limit of normal; ^$^Root mean squared error.

**Figure 2.**
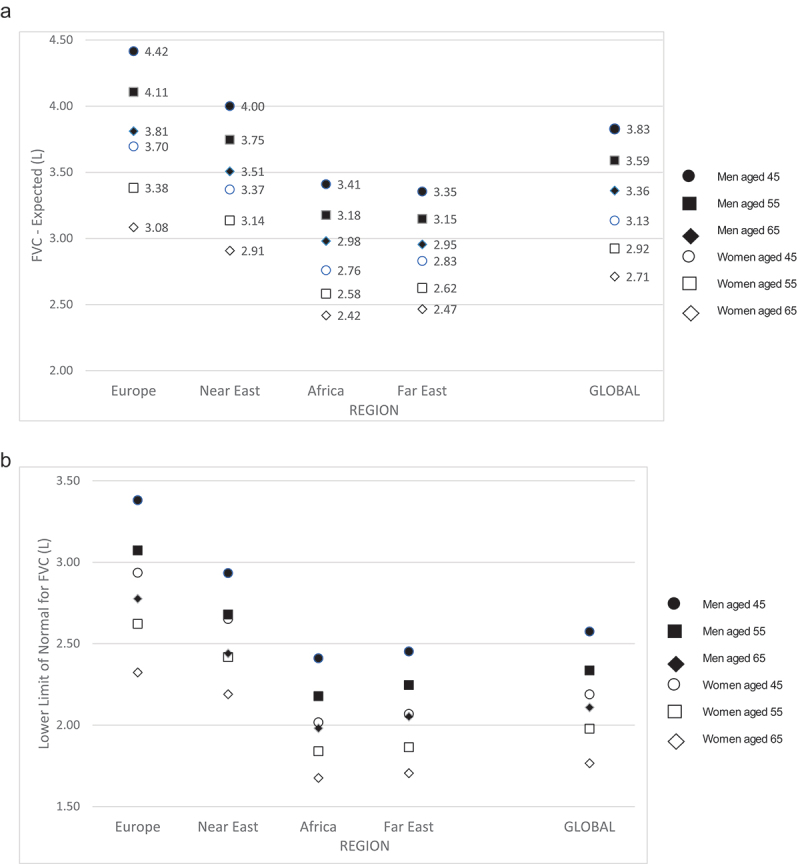
a) Expected values (50th centile) and b) lower limit of normal (5th centile) by age, sex and region for 1.65 m person. Expected values for a 1.65 m person by age, sex and region (m: metre; FVC: forced vital capacity; LLN: lower limit of normal). b) Lower limit of normal for forced vital capacity (FVC) for a 1.65 m tall person by age, sex and region.

Figure 2 shows the expected values of FVC (a) and the lower limit of normal (5^th^ centile) (b) for men and women of 1.65 m height by age and region. People of the same sex and height living in “Africa” or the “Far East” have expected values of FVC and lower limits of normal that are lower at age 45 than people living in “Europe” or the “Near East” have at 65 years.

Table 4 shows results from a multi-level model of the FEV_1_/FVC ratio against age and height^2^, grouped by site within each region, and the root mean squared error for each analysis. Although there is statistically significant variation, this is very small and unlikely to be clinically relevant. The lower limit of normal for a male or female aged 65 years and height 1.65 m is close to 0.68 (Table 5).

**Table 4.** Regional variation for FEV_1_/FVC ratio, showing regression coefficients from multi-level models with the root mean square error for the estimated value (50th centile) and lower limit of normal (LLN) (5th centile).

Region	Constant* (Expected value)	Constant* (LLN**)	Age	Height^2^	rootMSE^$^
**Men**					
Europe	0.83	0.74	−0.0023	−0.000009	0.05498
Near East	0.84	0.75	−0.0026	−0.000012	0.05801
Africa	0.84	0.72	−0.0027	−0.000007	0.06949
Far East	0.83	0.73	−0.0023	−0.000007	0.06478
Global	0.84	0.73	−0.0025	−0.000009	0.06392
**Women**					
Europe	0.84	0.75	−0.0022	−0.000025	0.05469
Near East	0.84	0.74	−0.0019	−0.000028	0.06020
Africa	0.83	0.73	−0.0020	−0.000011	0.06406
Far East	0.83	0.73	−0.0018	−0.000006	0.06398
Global	0.84	0.74	−0.0020	−0.000017	0.06020

*Constant for a 40-year-old, 1.40 m tall; ^$^Root mean squared error; **LLN: lower limit of normal (5^th^ centile).

**Table 5.** Expected and LLN values for the FEV1/FVC ratio by sex, age and region for a 1.65 m person.

	Expected values			Lower Limit of Normal		
	Men					
REGION	Age 45	Age 55	Age 65	Age 45	Age 55	Age 65
EUROPE	0.82	0.80	0.78	0.72	0.70	0.68
NEAR EAST	0.82	0.80	0.78	0.73	0.70	0.68
AFRICA	0.82	0.79	0.77	0.70	0.68	0.65
FAR EAST	0.82	0.80	0.78	0.71	0.69	0.67
GLOBAL	0.82	0.80	0.78	0.71	0.69	0.67
	Women					
EUROPE	0.83	0.79	0.77	0.72	0.70	0.68
NEAR EAST	0.83	0.80	0.78	0.73	0.71	0.69
AFRICA	0.82	0.80	0.78	0.71	0.69	0.67
FAR EAST	0.83	0.80	0.79	0.72	0.70	0.68
GLOBAL	0.83	0.80	0.78	0.72	0.70	0.68

## Discussion

In this large multinational, observational study, we found both between- and within-regional variations in the relation of FVC to age, sex and height. The small variation found for the FEV_1_/FVC ratio is likely to be of little clinical relevance.

PCA identified four groups of centres. These were clearly demarcated, particularly among the women. The naming of them assumes that these are predominantly due to geographical variation. The PCA placed the high-income countries with a similar socio-economic and environmental background, including lifestyle, in the same grouping as the European centres. Abbreviating the title of the group to “European” is not geographically correct but it is hard to think of an alternative that is as succinct. The PCA also placed the two Caribbean centres with those of Sub-Saharan Africa, which may be surprising given that Trinidad and Tobago is a high-income country with a high proportion of its population descended from South Asian migrants. It may also be counterintuitive to class all the centres in south, south-east and east Asia in one group, but this is the finding of the current analysis. With more data from more centres, this categorisation might change, but for the moment, the general category of “Far East” seems adequate.

Riyadh in Saudi Arabia was the only clear exception, being placed in the “Far East” group for both men and women. The only other exception was Tirana in Albania, which, for women, was closer to the “Near Eastern” group of sites than to the rest of the “European” group, though it was compatible with being a member of either group. The diagram for men is not quite as clear cut as that for women, but this can largely be explained by the small sample size in some of the sites such as Krakow (Poland) and Guangzhou (China). Where the sample sizes are small, this may represent not just less precision but also a potential selection bias where, for instance, a large number of smokers has had to be excluded. The large number of missing men due to high smoking rates in some sites is a particular problem. We have re-run the PCA of the FVC for men and women without excluding smokers and those with respiratory symptoms and diagnoses (Figure S1). This separates the four groups more clearly (again with the exception of Riyadh (Saudi Arabia)), a result that reflects the relatively small impact of smoking on the FVC.

Examining the associations between the FVC and age, sex and height shows that these associations vary widely. The coefficients for the constant were particularly low in the “Far East” and the coefficients for height^2^ were particularly low in Africa.

None of the geographical associations should be interpreted as demonstrating an ethnic determinism as we know that lung function has improved over time in Europe,^17–19^ and might well do so in other parts of the world in the future. We have used geographical terms to emphasise the probable importance of environmental determinants.

The coefficients for age were relatively low, showing a loss of about 38 and 32 mL/year in Europe, 25 and 26 mL/year in the Near East, 26 and 18 mL/year in Africa and 23 and 25 mL/year in the Far East, for men and women, respectively. The low values may be explained by the relatively young age particularly outside Europe, and this may also explain the lack of significance in the effects of age^2^. Changes with age in a cross-sectional survey will also reflect changes in lung function across generations. In Europe, lung function has been increasing across the generations, and this will appear as a greater fall with age in a cross-sectional study.^17,19^

The finding of significant variation in the height coefficients between regions differs from the finding of Kieffer et al. of similar height coefficients between ethnic groups in the USA,^15^ but our findings are associated with a much larger geographic spread. The variation in the coefficients for the constant is similar to the variation reported by Kieffer et al. between African Americans and other ethnic groupings.

That there are differences in lung function between different parts of the world is not surprising. That there is such a clear distinction between the four regions based only on the relation of the FVC to age and height is perhaps more remarkable. The differences between “Africa” and the “Far East” and “Europe” are larger than the differences generally quoted in Europe between indigenous groups and migrants from countries in “Africa” and the “Far East”, which suggest an approximately 10–15% lower FVC in people of African or South Asian descent.^3,20^ The results here suggest that the FVC is around 30% lower in “Africa” and the “Far East”. The difference could be explained by the greater likelihood for the physically fit to migrate, and possibly by the improved social and environmental conditions in Western countries.

## Clinical implications

Local “normal” values are primarily of value in the diagnosis of restrictive disorders. In this case, the natural comparator is a local person who has no smoking history, no other respiratory diagnosis and no symptoms. Although there are clear variations in “normal” lung function by region, it is important to note that there are also wide variations within each region. In addition, it should be noted that the residuals associated with the regression were not all normally distributed. All of which strongly argues that whatever guidelines are used, they need to be used understanding that expected values and lower limits of normal are never precise quantities.

The variation of normal values for the FVC between sites must not be interpreted as indicating differences in optimal lung function between regions. Data from the USA on differences in outcome between European Americans and African Americans suggest that the same lung function in European Americans and African Americans is associated with the same outcome, including mortality^6,^^7,^^8,^^9^ and that the lower “normal” values of FVC found in African Americans should not, therefore, be regarded as optimal. When assessing prognosis (severity), a single standard should therefore be used everywhere, either the global standard proposed by Bowerman^10^ or the more aspirational standard derived from the NHANES study in the USA.^1^ This conclusion is, however, based on information from the USA alone. Whether these findings are relevant beyond the USA has yet to be established.

There is no need for local equations when diagnosing obstructive disease. For this, the FEV_1_/FVC ratio is the relevant measurement, and a low FEV_1_ without reference to the FVC should not be interpreted as indicating obstruction. Fortunately, the “normal” values of the FEV_1_/FVC ratio do not vary substantially by region.

## Limitations

The validity of our findings depends on the selection of places and individuals. We aimed at a purposeful sample that included numbers in proportion to the cubed root of populations in the Global Burden of Disease regions. Latin America was not included as they had a separate study (PLATINO). We were not entirely successful with far fewer individuals, for instance, from East Asia and no centres from Oceania. The places were also selected for the presence of a Principal Investigator with an interest in the topic and a local team capable of undertaking the project. The size of each sample was selected so as not to allow extreme bias in the populations sampled.

Any survey of lung function is heavily dependent on the quality of the data, specifically the spirometry. Individual quality control was built into the BOLD study from the beginning with training of the technicians at each site. All individual tests were reviewed centrally and those technicians showing poor quality were retrained or moved to other work. One feature that is very hard to determine is the completeness of the blow. The end of the blow can be assessed but the completeness of the inspiration at the beginning of the test can only really be assessed by the technician at the time of the test. However, if this had been a major source of bias it would have to have affected all technicians in a region, and this seems unlikely.

## Conclusion

There are wide geographic variations in FVC among “normal” non-smoking individuals. These variations are not seen for the FEV_1_/FVC ratio. The low levels of FVC in some regions should not be considered optimal as they may well be associated with increased mortality.

## Supplementary Material

Supplemental Material
