# Supplementary material for: Geographical variation in lung function: Results from the multicentric cross-sectional BOLD study
Source: Pulmonology. 2024 Dec 6;31(1):2430491. doi: 10.1080/25310429.2024.2430491 (PMC11627206; doi:10.1080/25310429.2024.2430491)
Supplement: Supplemental Material [file TPUL_A_2430491_SM6261.docx]

**Figure S1.** Principal Component Analysis of FVC, age, age^2^ and height^2^ for women (A) and men (B), including ever smokers and those who report respiratory symptoms or diagnoses.

**A**

**B**
